# Strategies to Increase Professional Interpreting in Clinical Settings: A Systematic Review

**DOI:** 10.1001/jamanetworkopen.2025.21492

**Published:** 2025-07-17

**Authors:** Colleen K. Gutman, Christina R. Rojas, Lauren Waidner, Ronine L. Zamor, Emily A. Hartford, Desiree Yeboah, Elyse N. Portillo, K. Casey Lion, Elisabeth Nylander, Maria E. Garcia

**Affiliations:** 1 Department of Emergency Medicine, University of Florida College of Medicine, Gainesville; 2 Department of Pediatrics, University of Florida College of Medicine, Gainesville; 3 Division of Emergency Medicine, Ann & Robert H. Lurie Children’s Hospital of Chicago, Chicago, Illinois; 4 Herbert Wertheim College of Medicine, Florida International University, Miami; 5 Department of Pediatrics, Emory University School of Medicine, Atlanta, Georgia; 6 Department of Emergency Medicine, Emory University School of Medicine, Atlanta, Georgia; 7 Department of Emergency Medicine, Children’s Healthcare of Atlanta, Atlanta, Georgia; 8 Department of Pediatrics, University of Washington School of Medicine, Seattle Children’s Hospital, Seattle; 9 Division of Pediatric Emergency Medicine, Baylor College of Medicine, Texas Children’s Hospital, Houston; 10 Seattle Children’s Hospital and Research Institute, Seattle, Washington; 11 Department of Medicine, University of California, San Francisco; 12 Department of Epidemiology and Biostatistics, University of California, San Francisco

## Abstract

**Importance:**

Clinicians frequently communicate without professional interpreters when caring for patients who use languages other than English for medical care, despite evidence that such partnering improves health outcomes.

**Objective:**

To systematically review the evidence for targeted behavior change strategies to increase professional interpreting in medical settings.

**Evidence Review:**

The CINAHL, Embase, PubMed, Ovid MEDLINE, and Web of Science databases were searched on July 28, 2023, and September 2, 2024, for English-language peer-reviewed articles assessing all interventional and observational studies that included outcomes related to professional interpreting in medical settings. Two reviewers conducted each screening stage. The study design, implementation strategies, sample, and outcomes were extracted, and standardized criteria were applied to assess risk of bias. The data were synthesized according to the Capability, Opportunity, Motivation–Behavior (COM-B) model to map implementation strategies to behavior change targets. The Preferred Reporting Items for Systematic Reviews and Meta-Analyses (PRISMA) reporting guideline was followed.

**Findings:**

Of the 17 952 records identified, 40 were eligible for inclusion, representing 39 interventions. Most of the 39 studies used a pre-post design (21 [54%]) or quality improvement methodologies (15 [38%]). Heterogeneity in implementation strategies and outcome ascertainment precluded meta-analysis. Most studies had risk of bias due to unmeasured confounding; only 7 (18%) had low risk of bias. In addition, most studies (37 [95%]) described an increase in at least 1 professional interpreting measure. Ten studies (26%) assessed a single implementation strategy, and the 29 remaining studies (74%) assessed an intervention bundle with multiple strategies. Interventions frequently included strategies that provided clinician education (20 [51%]), increased clinician access to professional interpreting (18 [46%]), implemented standardized policies and procedures for professional interpreting (12 [31%]), and involved modifications to the electronic health record (14 [36%]). Mapped to the COM-B model, implementation strategies targeted clinician capability (32 interventions [82%]), opportunity (28 [72%]), and motivation (22 [56%]).

**Conclusions and Relevance:**

In this systematic review of targeted behavior change strategies to increase professional interpreting for clinician communication with patients using languages other than English for medical care, a wide variety of implementation strategies with high heterogeneity in outcome assessment were identified. Given clear improvement in patient health outcomes, there is a critical need to understand the discrete impact of specific strategies and how best to measure outcomes to inform efforts to increase professional interpreting in health systems.

## Key Points

**Question:**

What is the evidence for targeted behavior change strategies on clinician decisions to partner with professional interpreters when caring for patients who use languages other than English?

**Findings:**

This systematic review included 40 articles representing 39 interventions. High heterogeneity in targeted behavior change strategies and outcome ascertainment was identified among the 39 interventions seeking to increase professional interpreting for clinician communication with patients who use languages other than English when seeking medical care.

**Meaning:**

These findings suggest that to address the implementation gap in professional interpreting, there remains a need for rigorous efforts to understand the discrete impact of specific implementation strategies and how best to measure outcomes.

## Teaser

This systematic review evaluates targeted behavior change strategies to increase partnering with professional interpreters when caring for patients using languages other than English for medical care.

## Introduction

Language barriers in the US health care system contribute to health inequities faced by patients who use languages other than English (LOE) when seeking medical care.^1,2,3^ Professional interpreting by qualified interpreters, provided in person or remotely (via telephone or video) when there is discordance between clinician and patient language, improves health outcomes.^4,5^ Although it is an unfunded mandate, Title VI of the Civil Rights Act of 1964 requires that all entities receiving federal funding provide free professional interpreting.

Clinicians’ decisions of whether to partner with a professional interpreter for communication with patients who use LOE for medical care are influenced by individual factors (eg, concerns about the skill and time required for, and anticipated benefit of, professional interpreting) and system factors (eg, difficulty accessing professional interpreters).^6^ With these multilevel barriers, clinicians fail to partner with professional interpreters during an estimated 31% to 88% of clinical encounters with patients who use LOE for medical care.^7,8,9,10,11,12,13,14^ Instead, clinicians may communicate through ad hoc interpreters (eg, bilingual family members) or with their own nonproficient language skills.^6^ These approaches risk miscommunication and may result in patient harm.^15,16^

Suboptimal partnership with professional interpreters, despite evidence of benefit, is a critical implementation gap.^17^ Strategies implemented across hospitals and health systems to support clinicians in partnering with professional interpreters are few and limited.^18^ Those tested in smaller settings may not have the reach to drive system-wide changes, but they may collectively provide evidence to support organizational change. To identify and ultimately support the widespread adoption of strategies that may address this gap, we systematically reviewed the evidence for targeted behavior change strategies on clinician decisions to partner with professional interpreters when caring for patients using LOE in medical settings.

## Methods

This systematic review was registered with PROSPERO (CRD42023454738). We followed the Preferred Reporting Items for Systematic Reviews and Meta-Analyses (PRISMA) reporting guideline.

### Terminology

A wide range of terminology is used to describe patients using LOE, the work of professional interpreters, and clinicians who interact with patients. To be consistent and person centered, we use the following terminology while recognizing that there are limitations to each.^19,20^ We use *patient using LOE* to refer to patients who use LOE for medical care.^19,20^ We use *professional interpreting* to describe the work done by qualified professional interpreters. We use *clinician* to refer to medical professionals, including physicians, advanced practice professionals, nurses, and other clinical staff.

### Search Strategy and Data Sources

We assessed English-language peer-reviewed articles for behavior change strategies affecting clinicians’ decisions to partner with professional interpreters when caring for patients using LOE in medical settings. All interventional and observational study types that included outcomes related to professional interpreting were included. Abstracts, commentaries, protocols, and systematic reviews were excluded, as were investigations in nonmedical settings. A biomedicine research librarian (E.N.) developed search terms based on key concepts by looking at words in titles, abstracts, and subject indexing of known eligible studies. We validated a draft PubMed search by testing whether it could identify the relevant studies, which we then adapted to the syntax and subject headings of the other databases and platforms. The searches were limited to English, but not by publication date or study type. We searched the following databases on July 28, 2023, and September 2, 2024: CINAHL, Embase, Ovid MEDLINE, PubMed, and Web of Science. Search strategy details are provided in the eMethods in Supplement 1.

### Study Selection

Duplicate records were removed using an automated tool in the Systematic Review Accelerator (Bond University Institute for Evidence-Based Healthcare); remaining records were uploaded into the Covidence web-based tool (Covidence). We conducted 2 stages of screening: (1) title and abstract review and (2) full-text review. At each stage, items were reviewed by the first author (C.K.G.) and a second independent reviewer (C.R.R., L.W., R.L.Z., E.A.H., D.Y., E.N.P., K.C.L., or M.E.G.). Discrepancies were reviewed to reach consensus; the senior author (M.E.G.) adjudicated unresolved differences.

### Data Extraction

We extracted data (details of the study design, implemented strategies to enact behavior change, sample, and outcomes) from included studies into a structured data collection tool in Covidence. Two reviewers (C.K.G. and either C.R.R., L.W., D.Y., or M.E.G.) independently extracted data from 10% of included studies and demonstrated acceptable interrater reliability (κ > 0.6) on assessed data elements. A single reviewer (C.K.G.) extracted data from the remaining included studies.

### Quality Assessment

Due to heterogeneity in study design, there was no single suitable tool for assessing study quality. To address this, we adapted a checklist from several existing tools, including the Methodologic Index for Non-Randomized Studies,^21^ the Quality Improvement Minimum Quality Criteria Set,^22^ and the JBI Critical Appraisal Tools for Cross-Sectional and Quasi-Experimental Studies.^23^ We chose these tools because they applied to the most frequent study designs in our dataset and assessed relevant potential sources of bias. The final tool consisted of 11 items (eTable in Supplement 1). Two reviewers (C.K.G. and C.R.R.) independently applied the tool and reviewed and resolved discrepancies; the senior author (M.E.G.) adjudicated unresolved differences.

### Data Analysis

Due to the heterogeneity in outcome ascertainment as well as variation in the types of implementation strategies assessed, we were unable to conduct a meta-analysis on professional interpreting outcomes.^24^ Instead, we conducted a qualitative analysis and framework synthesis of data, guided by the Theoretical Domains Framework (TDF) mapped to the Capability, Opportunity, Motivation–Behavior (COM-B) model for behavior change (Table 1).^25,26,27^ The TDF and the COM-B provide a framework for understanding how strategies target individual behaviors and a basis for strategically designed behavior change strategies.^25,26,27^ This framework defines 3 components necessary for behavior change: individuals must have the knowledge and skills to (1) be capable of the behavior, (2) work within an environment that presents them the opportunity to use the behavior, and (3) have the motivation to engage in the behavior.^25^

**Table 1.** TDF Mapped to the COM-B Model of Behavior Change in Relation to Professional Interpreting

COM-B component and TDF domain	Examples of barriers and facilitators related to professional interpreting
Capability	
Knowledge	Knowledge of how and when to partner with professional interpreters
Skills	Ability to effectively partner with professional interpreters
Memory, attention, decision processes	Cognitive overload of additional tasks during clinical care affecting partnership with professional interpreters
Behavior regulation	Habits around communicating with or without professional interpreting
Opportunity	
Environmental context and resources	Difficulty finding remote professional interpreting device
Social influences	Norms around professional interpreting (everyone else does it, or no one else does it)
Motivation	
Beliefs about capabilities	Confidence in ability to effectively partner with professional interpreters
Beliefs about consequences	Concerns about time requirement and about quality of interpreting; conceptual knowledge of why professional interpreting is important
Social and professional role/identity	Self-perceived duty to provide high-quality care with professional interpreting
Emotions	Guilt when getting by without professional interpreting; stress leading clinicians to skip professional interpreting
Optimism and pessimism	Low or high anticipated benefit of professional interpreting
Goals	Desire to increase professional interpreting behaviors
Intentions	Intent to increase professional interpreting behaviors
Reinforcing behavior	Satisfaction with experience partnering with professional interpreters

Abbreviations: COM-B, Capability, Opportunity, Motivation–Behavior; TDF, Theoretical Domains Framework.

The target behavior was clinician partnering with professional interpreters for communication with patients using LOE. We further assessed 2 aspects of outcome measurement: (1) the outcome source (eg, administrative data or patient report) and (2) the type of outcome (eg, per encounter or during specific interactions).

For each intervention, we determined whether there was a single implementation strategy to enact behavior change (ie, a single targeted behavior change technique) or a bundle of 2 or more implementation strategies. We mapped each implementation strategy to COM-B components, guided by the TDF (Table 1).^25,26,27^

## Results

We retrieved 17 952 records through database searching. After duplicates were removed, 7934 records underwent title and abstract screening; 187 records underwent full-text review (eFigure in Supplement 1). A total of 40 articles detailing 39 interventions were included in this review (Table 2).^28,29,30,31,32,33,34,35,36,37,38,39,40,41,42,43,44,45,46,47,48,49,50,51,52,53,54,55,56,57,58,59,60,61,62,63,64,65,66,67^ Study details are presented in Table 3. Most studies used a pre-post design (21 [54%]) or quality improvement methodologies (15 [38%]); 2 (5%) were randomized trials and 1 (3%) was a case study. Heterogeneity in implementation strategies and outcome ascertainment precluded meta-analysis. Herein, we summarize our findings related to study quality, professional interpreting outcomes, implementation strategies to enact behavior change, and COM-B and TDF behavior change targets.

**Table 2.** Characteristics of Included Studies

Characteristic	No. (%) of studies (n = 39)^a^
Country	
Australia	4 (10)
Canada	2 (5)
Switzerland	3 (8)
US	30 (77)
Clinical setting	
Single emergency department	11 (28)
Single inpatient unit	7 (18)
Single outpatient clinic	1 (3)
Multiple inpatient units, single hospital	5 (13)
Multiple outpatient units, single hospital	1 (3)
Entire hospital	4 (10)
Entire health system	6 (15)
Multiple sites	4 (10)
Study type	
Pre-post intervention design, nonrandomized	21 (54)
QI initiative with PDSA cycles	15 (38)
Randomized trial	2 (5)
Case study	1 (3)
Intervention components	
Single-strategy intervention	10 (26)
Intervention bundle	29 (74)
Intervention duration	
≤6 mo	6 (15)
7-12 mo	4 (10)
1-2 y	12 (31)
>2 y	13 (33)
Not specified	4 (10)
Source of professional interpreting outcome data^b^	
Administrative data	
Linked to patient encounters	5 (13)
Not linked to patient encounters	10 (26)
Patient reported	6 (15)
Clinician reported	
EHR documentation	8 (21)
Related to specific patient encounters	3 (8)
Reflection on usual practice	8 (21)
Not specified	5 (13)
Type of professional interpreting outcome data^b^	
Always occurring for clinical care (patient reported)	2 (5)
At least once per visit (outpatient) or per day (inpatient)	15 (38)
Instances per patient-day	6 (15)
During specific interaction types	8 (21)
Increase in at least 1 measure of professional interpreting	37 (95)

Abbreviations: EHR, electronic health record; PDSA, Plan-Do-Study-Act; QI, quality improvement.

^a^ One study reported results in 2 included articles^28,29^ and is presented as 1 study.

^b^ Studies could include more than 1 outcome type.

**Table 3.** Setting, Implementation Strategy, Sample, and Outcome Details of Included Studies by Outcome Type

Source	Setting	Implementation strategy details, mapped to COM-B^a^	Sample	Outcomes
**Professional interpreting always occurred for clinical care or when needed**				
Lion et al,^41^ 2015	US academic pediatric hospital, 2010-2012	Education: 10-min interactive sessions at required physician, nurse, and staff meetings (C, M) Access: dual-handset telephones with single-touch access to language line at every patient bedside (C, O) EHR: pop-up alert recommending telephone interpreting for brief communications (C, O, M)	Convenience sample of patients using LOE (n = 185 [64.6% participation rate] before, 117 [78.5%] during, and 203 [70.0%] after)	Increase from 53.3% to 71.8% (PR)
Douglas et al,^61^ 2024	US academic pediatric ED, 2021-2022	Education: 20-min presentation to physicians and nurses at staff meeting; email to residents at start of rotation (C) Access: increased video interpreting devices (from 1 to 3 in ED) (O) Policies: standardized assessment of language and interpreter need at check-in (“How well do you speak and understand English?”) (C, O, M)	Random sample of patients using LOE (n = 312 [51.1% response rate])	No change from 54% (PR)
**Professional interpreting occurred at least once per visit or per inpatient day**				
Standiford et al,^33^ 2009	US academic hospital, 2006-2008	Policies: calls to patients using LOE to confirm language and schedule interpreter (outpatient [C, O]); patient visits linked to interpreter system (O); standard script to identify language at check-in (“What language would you prefer to use to speak to the doctor or nurse?”) (C, O, M) EHR: language field changed from “primary language” to “language for care” (M); prompt for language assessment at check-in (C, O, M) Other: designated nurse champion (O); daily rounds on patients using LOE by an in-person interpreter to ensure staff and families were aware of interpreter services (C, O, M)	Patients in institution diabetes registry (n = 9931)	Increase from 19% to 83% (L)
Buser et al,^56^ 2022	Swiss academic pediatric ED, 2021	Education: 1-h online module and 2-h in-person training (all health care workers) (C, M); reminders in meetings and newsletters (C, M) Policies: standardized pathway and scripts for (1) identifying language and interpreter need in triage, (2) offering use of interpreters during clinical care, and (3) ordering and documenting professional interpreting by ED unit clerk (C, O, M)	Consecutive ED visits by patients using LOE (n = 127 before vs 135 after)	No significant change from 11.0% to 14.8% (L)
Hartford et al,^57^ 2022	US academic pediatric ED, 2019-2021	Policies: policy promoting telephone communication in place of in-person interactions during the COVID-19 pandemic (O)	Consecutive ED visits by patients using LOE (n = 1934 before vs 1506 after)	Increase from 59% to 73% (L)
Hartford et al,^60^ 2023	US academic pediatric ED, 2015-2021	Education: education during required physician and nurse staff meetings (C, M) Access: Spanish in-person interpreter present in a high-visibility location in the ED during high-need times (C, O); video interpreting units in all patient rooms (C, O) Policies: standard script to assess language at check-in: “What language would you like for care today?” followed by “Can we provide free interpretation in [language of choice]?” (C, O, M) EHR: icon displaying language on patient list (C, O, M) Other: interpreter use data displayed via central dashboard and through emails and presentations (O, M); language sign on doors to patient rooms (C, O, M)	Consecutive ED visits by patients using LOE (n = 33 793)	Increase from 53% to 82% (L)
Gupta et al,^59^ 2023	US academic pediatric ED, 2020	Education: emails to clinicians (C) Policies: standard script to assess language at triage: “Do you speak any languages other than English?” followed by “Our hospital [CHOP (Children’s Hospital of Philadelphia)] has free and quickly accessible interpreter services. What language would you like us to speak to you in?” (C, O, M) EHR: script for language assessment built into ED triage tool (C, O, M); icon displaying language on patient list (C, O, M); pop-up alert about language and interpreting when opening medical record for patients using LOE (C, O, M); template to document professional interpreting built into all clinician notes for patients using LOE (C, O, M)	Consecutive ED visits (n = 54 922)	Increase from 77% to 86% (L)
Taira et al,^55^ 2021	US academic ED, 2016-2018	Education: physician grand rounds (C, M); on-shift teaching sessions (C, M); screensaver reminders (C, M); quick reference cards (C) Access: increased internet bandwidth (O); telephones with speaker capability in patient rooms with sticker showing interpreter services telephone number (C, O) EHR: icon indicating interpreter need on patient list (C, O, M) Other: clinical and administrative champions (O)	Consecutive patients using LOE who requested an interpreter during defined 48-h periods (n = 110 before vs 134 after)	Increase from 15.5% to 29.6% (clinician documented in EHR)
Martinez et al,^51^ 2021	US academic pediatric ED, 2017-2020	EHR: icon displaying language on patient list (C, O, M); 3-click form for documenting professional interpreting (C, O, M)	Consecutive patients who use Spanish for medical care (n = 988 before vs 710 after)	Increase from 35.7% to 64.5% (clinician documented in EHR)
Rajbhandari et al,^53^ 2021	US academic pediatric inpatient unit, 2017-2019	Education: 30-min interactive sessions with physicians and nurses (C, M); emails to residents at start of rotation (C, M); required annual online module for nurses (C, M); education for registration staff (C) Access: increase number of video interpreting devices with designated storage space on each unit (O) Policies: language identified at registration and verified by medical team at admission (C, O, M); daily list of rounding times for patients using LOE provided to interpreters and physicians (C, O) EHR: language access navigator for standardized documentation of professional interpreting (C, O, M); computerized order entry to request interpreting (C, O) Other: individual professional interpreting data added to existing physician incentive plan (M)	Consecutive admitted patients using LOE (n = 64 before, 251 during, and 93 after)	Increase from 64% to 98% (clinician documented in EHR)
Behairy et al,^58^ 2023	US academic pediatric inpatient unit, 2020	Education: email to physicians and nurses (C, M); flyers in clinician work areas (C); conference for residents (C, M) Access: dual-handset telephones at the bedside of every patient using LOE (C, O) EHR: standardized phrase for documentation of professional interpreting entered into the patient medical record at clinician discretion (C, O, M); column indicating language on patient list (C, O, M) Other: informational brochures handed to patients at admission (C, O, M)	Convenience sample of admitted patients using LOE (n = 91 before vs 148 after)^b^	Increase from 0% to 59% (clinician documented in EHR)
Jaradeh et al,^63^ 2024	US academic ED, 2021-2023	EHR: standardized phrase for documentation of professional interpreting entered into the patient medical record at clinician discretion (phase 1 [C, O, M]); hard stop requiring documentation of professional interpreting (phase 2 [C, O, M])	Randomly selected patients using LOE (n = 91 before, 809 in phase 1, and 646 in phase 2)	Increase from 35% to 43% in phase 1 to 97% in phase 2 (clinician documented in EHR)
Magana-Soto et al,^65^ 2024	US academic pediatric inpatient unit, 2020-2022	Education: sessions for physicians and nurses (C, M); physician grand rounds (C, M) Access: in-person Spanish interpreter present during high-need times (C, O) Policies: rounding schedule provided to in-person interpreter (C, O) EHR: assessment of language and interpreter need added to nursing intake module (C, O, M); standardized documentation of interpreting during rounds (by in-person interpreter) (M) Other: interpreter request cards for patients (C, O, M); central dashboard displaying interpreter use data (O, M)	Patients who used Spanish for medical care and were admitted on a weekday (n = 442)	Increase from 39.4% to 51.9% (adjusted odds post intervention, 1.64 [clinician documented in EHR])
Douglas et al,^61^ 2024	See earlier study description	See earlier study description	Consecutive patients using LOE	Increase from 20% to 32% (clinician documented in EHR)
Karliner et al,^64^ 2024	US academic outpatient clinic, 2014-2017	Access: introduction of video interpreting (O) Other: oral Spanish proficiency testing (M)	Consecutive patients who used Spanish or Mandarin for medical care and received care from language-discordant or partially concordant clinicians (n = 698)^b^	Increase from 57.4% to 65.9% (PR)
Mazor et al,^31^ 2002	US academic pediatric ED, 2000	Other: 10-wk medical Spanish course (2 h/wk); mock interviews followed by a written test to assess comprehension of key history elements, completed before and after the course (M)	Consecutive patients who use Spanish for medical care and who received care from a participating physician (n = 85 before vs 58 after)^b^	Decrease from 55% to 29% (CR)
Bischoff et al,^32^ 2003	Swiss academic outpatient clinic, 1999-2000	Education: four 1- to 2-h workshops over 2 mo (C, M)	Convenience sample of patients who used a language other than French for medical care (n = 161 before vs 249 after)^b^	Increase from 46% to 67% (NS)
Regenstein et al,^36^ 2012	10 US hospitals, 2007-2008	Varied by site; details not specified	10 nonfederal acute care hospitals	Increase from 34% to 53% (NS)
Yelland et al,^42^ 2017	Australian inpatient unit (labor and delivery), 2014-2015	Education: in-service education for all staff (C); brochure (C); flyer posted in staff restrooms (C)	Consecutive patients who use a language other than English for medical care (n = 74 before vs 72 after)	Increase from 28.4% to 63.5% (NS)
**Professional interpreting encounters per day or admission**				
Stolk et al,^30^ 1998	Australian inpatient unit (psychiatric), 1995	Education: four 90-min sessions; posted flyers (C, M)	Consecutive inpatient-bed days by patient using LOE (n = 429 before vs 400 after)	Increase from 0.02 to 0.1 per day (NL)
Tuot et al,^37^ 2012	US academic inpatient unit, 2007-2008	Education: sessions for nurses and instructional pocket cards for physicians (C) Access: dual-handset telephones with single-touch access to language line at every patient bedside (C, O)	Total telephone interpreter encounters^c^	Increase from 1.3 to 5.2 per admission (NL)
Lion et al,^41^ 2015	See earlier study description	See earlier study description	Consecutive inpatient-bed days by patient using LOE (n = 10 890 before, 3918 during, and 9472 after)	Increase from 0.38 to 0.58 (phone only) and from 0.96 to 1.47 (all interpreting) per day (NL)
Karliner et al,^43^ 2017	US academic inpatient unit, 2007-2010	Education: sessions for nurses and residents; instructional pocket cards for physicians (C) Access: dual-handset telephones with single-touch access to language line at the bedside of every patient using LOE (C, O)	Consecutive inpatient discharges by patients using LOE (n = 1963)	Increase from 1.7 to 7.9 (during) and 5.9 (post) per admission (mean length of admission 5.5 d [NL])
Narang et al,^47^ 2019	US academic oncology urgent care, 2014-2015	Access: app downloaded to clinician- or hospital-owned device with pretranslated medical phrases and close-ended questions, with a link to call out to the telephone interpreter service (C, O)	Total telephone interpreter encounters^c^	Increase from 0.15 to 0.26 calls per LOE visit (not sustained [NL])
Feister et al,^62^ 2025	US academic neonatal ICU, 2021-2022	Education: 30-min sessions for physicians and nurses (C, M); physician grand rounds (C, M) Access: Spanish in-person interpreter physically present, available on group text with clinicians for 2 h on weekdays during rounds (C, O); Spanish in-person check-in on night shift for night rounds (C, O) Policies: language added to patient handoff tool and daily safety huddles (O, M) EHR: language field added to daily progress note (C, O, M) Other: interpreter request card for patients (C, O, M)	Total interpreter encounters for patients who speak Spanish or Central American indigenous languages^c^	Increase from 0.2 to 1.0 per day (NL)
**Professional interpreting during specific clinical interactions**				
Tuot et al,^37^ 2012	See earlier study description	See earlier study description	Convenience sample of nurses (n = 127 before vs 122 after [65% participation rate]) and physicians (n = 96 before vs 78 after [67% participation rate])	Nurses: increase from 38% to 66%, 45% to 61%, and 62% to 77% (nurse admission, medications, and discharge [CR]); physicians: increase from 48% to 67% and 16% to 41% (before rounds, patient updates [CR])
Lee et al,^28^ 2017; Lee et al,^29^ 2018	US academic inpatient unit, 2012-2013	Education: sessions for nurses and physicians at staff meetings; article in online newsletter (C) Access: dual-handset telephones with single-touch access to language line at every patient bedside (C, O)	Consecutively enrolled patients who use Chinese or Spanish for medical care (discharge analysis: n = 94 before vs 95 after; and informed consent analysis: n = 84 before vs 68 after)^b^	Increase from 29.8% to 39.7% (informed consent for surgical procedures [PR]); no significant change from 15% to 7% (discharge education [PR])
Lopez-Bushnell,^50^ 2020	US academic hospital^d^	Education: 6 sessions for clinicians (C)	Convenience sample of admitted patients using LOE (n = 99)^b^	Increase from 56% to 74% (nurse explanation of medications, tests, laboratory results, diet, daily goals [PR]); increase from 51% to 69% (nurse communication about activities of daily living [PR]); increase from 61% to 77% (physician communications [PR])
Jaradeh et al,^63^ 2024	See earlier study description	See earlier study description	See earlier study description	Increase from 0% to 5% in phase 1 to 35% in phase 2 for procedures (clinician documented in EHR)
Trang et al,^67^ 2024	US academic operating room, 2022-2023	EHR: electronic consent form for generic informed consent prior to surgical procedures automatically populated in the patient’s language for care (Spanish, Chinese, Arabic, or Russian) (C, O, M); required documentation of professional interpreting when documenting informed consent (C, O, M)	Randomly selected patients using LOE (n = 1267 before vs 1016 after)	Increase from 56.9% to 83.9% for informed consent (clinician documented in EHR)
Cheston et al,^44^ 2018	US academic pediatric inpatient unit, 2014-2016	Policies: language added to patient handoff tool (O, M); standardized process for scheduling interpreters for rounds facilitated by medical students and unit clerks (C, O) Other: resident-team specific interpreting use data shared weekly by email (O, M)	Consecutive family-centered rounds encounters with patients using LOE (n = 614)	Increased from 0% to 63% (PR)
Ondusko et al,^52^ 2021	US academic pediatric inpatient unit^d^	Policies: comparison of 3 scripted questions asked on morning rounds: (1) “Would you like an interpreter?” (2) “A free hospital interpreter is available for you. Would you like an interpreter?” (3) “In what language do you prefer to receive your medical care?” (C, O, M)	Convenience sample of patients who use Spanish for medical care (n = 55)^b^	Overall, 82% (100% for preferred language, 82% for freely available, 64% for interpreter offer [CR])
Lion et al,^35^ 2012	3 US pediatric residency programs, 2010	Other: oral Spanish proficiency testing (M)	Pediatric residents from 3 residency programs (n = 76 [31.3% participation rate])	Decrease from 56% to 39% in report of comfort using Spanish in straightforward clinical scenarios (among physicians tested to be nonproficient in Spanish [CR])
**Ad hoc interpreting**				
Tuot et al,^37^ 2012	See earlier study description	See earlier study description	See earlier study description	Nurses: decrease from 37% to 18% (CR); physicians: decrease from 51% to 35% (CR)
Paradise et al,^48^ 2019	US health system, 2012-2018	Education: medical staff meeting presentation (C, M); brochure in physician orientation materials (C, M), 90-s videos in monthly emails (C, M) Access: increased video units and interpreter staffing to meet need (O); upgraded technical systems to bandwidth (O) Policies: policy prohibiting use of family and friends as interpreters (O, M) Other: patient-facing education about professional interpreting on screens in waiting rooms (C, O, M); regular meetings to review local data with participating sites (O, M)	Clinical sites in a regional health system with >100 encounters with patient using LOE per month (n = 27)	Increase from 41% to 59% of sites with ad hoc interpreters in fewer than 10% of encounters with patients using LOE (clinician documented in EHR)
**General practices around professional interpreting**				
Zuniga et al,^38^ 2013	US academic hospital, 2006 and 2011	Education: 30-min training for all staff; articles on institution intranet and sent by email (C, M)	Convenience sample of health science center students and faculty with patient contact (n = 333 after; before sample size unknown)^b^	Increase from 25.5% to 35.4% (“I use medical interpreters when I have a Spanish-speaking patient” [CR])
Hudelson et al,^39^ 2014	Swiss academic hospital^d^	Education: brief presentation during new staff orientation (C); brochures (C); video on hospital intranet (C, M); article in hospital newsletter (C, M); public events with music and food highlighting initiative (C, M) EHR: language field added (C, O, M) Other: reference nurse for all migrant care issues, including language services (O)	Randomly selected hospital staff (n = 1460 [51% participation rate] before vs 761 [19% participation rate] after)	Increase from 39.7% to 65.7% from 12.1% to 26.5% (use of in-person, telephone interpreter at least once in 6 mo [CR])
Dowbor et al,^40^ 2015	Canadian academic health system^d^	Access: coordinated bulk purchase of telephone interpreter services for entire organization (O)	Convenience sample of clinicians and staff from 30 of 34 participating clinical sites (n = 127)^b^	Decrease from 37% to 24%, 35% to 16%, 23% to 11%, and 11% to 4% (use of in-person, other clinicians, and other staff as interpreters; asking patients to bring their own interpreters [CR])
Kaur et al,^45^ 2019	Australian oncology nurses^d^	Education: four 30-min online modules (C, M)	Convenience sample of oncology nurses who completed both the before and after surveys (n = 53, of 108 completing at least 1 survey)^b^	Increase from 39% to 70% (“frequently” use medical interpreters for treatment, interventions, meetings, or other events [CR])
Berk et al,^49^ 2020	US academic ED, 2019	Other: oral assessment of Spanish skills through the Abbreviated Assessment of Health Literacy for Spanish Adults (M)	Convenience sample of emergency medicine clinicians (n = 50)^b^	Half of physicians who tested nonproficient were “more likely” to use professional interpreting after being told their results (CR)
**Overall professional interpreting**				
Karliner and Mutha^34^ 2010	US academic hospital^d^	Access: increased staff interpreters for video and telephone interpreting (O); centralized interpreter dispatch (O); enhanced IT setup for video conferencing (O)	Total interpreter encounters^c^	Increase 91.3%, from 3800 to 7270 per month (NL)
Zuniga et al,^38^ 2013	See earlier study description	See earlier study description	Total interpreter encounters^c^	Increase from 3941 to 88 282 per year (NL)
Marshall et al,^46^ 2019	US academic hospital, 2012-2018	Access: introduction of video remote interpretation devices (O)	Total interpreter encounters^c^	Increase from 15 000 to 100 000 per year (estimated from Figure 1 in Marshall et al) (NL)
Sharfuddin et al,^66^ 2024	Canadian academic hospital, 2019-2021	Education: 45-min sessions for physicians, nurses, and staff (C, M); 20-min huddles during clinical care (C); posted flyers (C) Access: introduction of video remote interpretation devices (3 in ED, 1 shared by general medicine units) (O); introduction of single-touch access to language line on all ED telephones (C, O) Other: posted flyers informing patients about interpreter availability with no associated cost (C, O, M)	Total interpreter encounters^c^	Increase from 3587 to 5159 min/mo (NL)
Saito et al,^54^ 2021	31 Australian outpatient clinics^d^	Varied by site; details not specified	General practice clinics (n = 17 randomized to intervention and 14 to control)	Increase from 101 to 174 professional interpreting encounters (control group with no change from 42 to 45 [NL])

Abbreviations: COM-B, Capability, Opportunity, Motivation–Behavior; CR, clinician reported; ED, emergency department; EHR, electronic health record; ICU, intensive care unit; IT, information technology; L, administrative data linked to specific patient encounters; LOE, languages other than English for medical care; NL, administrative data not linked to patient encounters; NS, not specified; PR, patient reported.

^a^ Categorized as targeting clinician capability (C), opportunity (O), or motivation (M).

^b^ Participation rate not reported.

^c^ Sample size not reported.

^d^ Time frame not reported.

### Study Quality

Seven studies (18%) had low risk of bias (Table 4).^28,29,31,53,56,57,64^ Twenty-seven studies (68%) had nonrandomized designs or did not report patient characteristics in preintervention and postintervention cohorts, resulting in a high risk of unmeasured confounding.^30,32,34,35,36,37,38,39,41,42,43,44,46,47,48,50,51,54,55,58,59,60,61,62,63,66,67^ Fourteen studies (35%) used convenience samples, did not report participation rates, or both, placing them at high risk of selection and nonresponse bias.^32,34,36,38,40,45,46,49,50,52,54,58,61,66^

**Table 4.** Details of Study Quality

Source	Clearly stated aim^a^	Participants and setting detailed^a^	Baseline interpreter access and use detailed^a^	Inclusion criteria defined^a^	Intervention described in detail^a^	Valid outcome measure^b^	Prospective data collection^c^	Consecutive patients^d^	Participation rate reported^e^	Similar participants in comparisons^f^	Analysis appropriate^a^
Behairy et al,^58^ 2023	Y	Y	Y	Y	Y	Y	Y	U	N	U	Y
Berk et al,^49^ 2020	Y	Y	N	Y	Y	N	NA	NA	N	Y	Y
Bischoff et al,^32^ 2003	Y	Y	Y	Y	Y	Y	Y	U	N	N	Y
Buser et al,^56^ 2022	Y	Y	Y	Y	Y	Y	Y	Y	Y	Y	Y
Cheston et al,^44^ 2018	Y	Y	Y	Y	Y	Y	Y	Y	Y	U	Y
Douglas et al,^61^ 2024	Y	Y	Y	Y	Y	Y	Y	N	Y	U	Y
Dowbor et al,^40^ 2015	Y	Y	N	N	Y	N	Y	N	N	Y	Y
Feister et al,^62^ 2024	Y	Y	Y	Y	Y	Y	Y	Y	NA	U	Y
Gupta et al,^59^ 2023	Y	Y	Y	Y	Y	Y	Y	Y	Y	U	Y
Hartford et al,^57^ 2022	Y	Y	Y	Y	Y	Y	Y	Y	Y	Y	Y
Hartford et al,^60^ 2023	Y	Y	Y	Y	Y	Y	Y	Y	Y	U	Y
Hudelson et al,^39^ 2014	Y	N	Y	Y	Y	N	Y	NA	Y	U	Y
Jaradeh et al,^63^ 2024	Y	Y	Y	Y	Y	Y	Y	Y	Y	U	Y
Karliner and Mutha^34^ 2010	Y	Y	Y	N	Y	N	U	Y	N	U	Y
Karliner et al,^43^ 2017	Y	Y	Y	Y	Y	Y	Y	Y	Y	U	Y
Karliner et al,^64^ 2024	Y	Y	Y	Y	Y	Y	Y	Y	Y	Y	Y
Kaur et al,^45^ 2019	Y	N	N	Y	Y	N	Y	NA	N	Y	Y
Lee et al,^28^ 2017	Y	Y	Y	Y	Y	Y	Y	Y	Y	Y	Y
Lee et al,^29^ 2018	Y	Y	Y	Y	Y	Y	Y	Y	Y	Y	Y
Lion et al,^35^ 2012	Y	Y	N	Y	Y	N	Y	NA	Y	Y	Y
Lion et al,^41^ 2015	Y	Y	Y	Y	Y	Y	Y	Y	Y	U	Y
Lopez-Bushnell,^50^ 2020	Y	Y	N	Y	Y	Y	Y	N	N	U	Y
Magana-Soto et al,^65^ 2024	Y	Y	Y	Y	Y	Y	Y	Y	NA	U	Y
Marshall et al,^46^ 2019	Y	Y	Y	Y	Y	Y	Y	Y	N	U	Y
Martinez et al,^51^ 2021	Y	Y	N	Y	Y	Y	Y	Y	Y	U	Y
Mazor et al,^31^ 2002	Y	Y	Y	Y	Y	Y	Y	Y	Y	Y	Y
Narang et al,^47^ 2019	Y	N	Y	Y	Y	Y	Y	Y	NA	U	Y
Ondusko et al,^52^ 2021	Y	Y	N	Y	Y	Y	Y	Y	N	Y	Y
Paradise et al,^48^ 2019	Y	Y	Y	Y	Y	Y	Y	Y	NA	U	Y
Rajbhandari et al,^53^ 2021	Y	Y	Y	Y	Y	Y	Y	Y	NA	Y	Y
Regenstein et al,^36^ 2012	Y	Y	Y	Y	N	U	Y	U	N	U	Y
Saito et al,^54^ 2021	Y	Y	Y	Y	N	N	Y	Y	N	U	N
Sharfuddin et al,^66^ 2024	Y	Y	Y	Y	Y	Y	Y	Y	N	U	Y
Standiford et al,^33^ 2009	Y	Y	N	Y	Y	Y	Y	Y	Y	Y	Y
Stolk et al,^30^ 1998	Y	Y	Y	Y	Y	Y	Y	Y	NA	U	Y
Taira et al,^55^ 2021	Y	Y	Y	Y	Y	Y	Y	Y	NA	U	Y
Trang et al,^67^ 2024	Y	Y	Y	Y	Y	Y	Y	N	NA	U	Y
Tuot et al,^37^ 2012	Y	Y	Y	Y	Y	Y	Y	Y	Y	U	Y
Yelland et al,^42^ 2017	Y	Y	Y	Y	Y	U	Y	U	Y	U	Y
Zuniga et al,^38^ 2013	Y	N	N	Y	N	N	Y	NA	N	U	Y

Abbreviations: N, no; NA, not applicable; U, unclear; Y, yes.

^a^ Response options were yes or no.

^b^ Response options were yes (if administrative data with an associated sample size or patient or clinician report at or near the time of a specific clinical encounter) or no (if clinician report of general practice patterns or if administrative data were presented without a denominator or sample size).

^c^ Response options were yes, no, unclear, or not applicable (for cross-sectional study designs).

^d^ Response options were yes, no, unclear, or not applicable (if outcomes were not related to patient encounters).

^e^ Response options were yes, no, or not applicable (for administrative data, select yes if linked to patient encounters or a total sample size is provided; select no if the data presented were a raw number of interpreting encounters without a denominator or sample size).

^f^ Response options were yes, no, or unclear.

### Behavior Change Outcome: Professional Interpreting

Thirty-seven studies (95%) described an increase in at least 1 measure of professional interpreting (Tables 2 and 3),^28,30,32,33,34,35,36,37,38,39,40,41,42,43,44,45,46,47,48,49,50,51,52,53,54,55,57,58,59,60,61,62,63,64,65,66,67^ with variation in outcome ascertainment. Seventeen studies (44%) assessed the proportion of patients using LOE who received professional interpreting at least once per visit or day.^31,32,33,36,42,51,53,55,56,57,58,59,60,61,63,64,65^ Professional interpreting was ascertained several ways, including through administrative data linked to discrete patient encounters (5 studies),^33,56,57,59,60^ EHR documentation (7 studies),^51,53,55,58,61,63,65^ patient report (1 study),^64^ clinician report (1 study),^31^ or an undefined source (3 studies).^32,36,42^ Fifteen studies showed an increase in professional interpreting.^32,33,36,42,51,53,55,57,58,59,60,61,63,64,65^ Six studies (15%) assessed professional interpreting events per LOE patient-day, all ascertained through system-level administrative data; all increased professional interpreting.^30,37,41,43,47,62^

Eight studies (21%) assessed professional interpreting in specific clinical interactions (eg, during medication administration or hospital discharge).^28,29,35,37,44,50,52,63,67^ In 4 of these studies, the implementation strategy targeted specific interactions (namely, professional interpreting during informed consent before procedures^63,67^ or during family-centered rounds^44,52^); professional interpreting increased in all of these. In the other 4 studies, implementation strategies were broadly focused across clinical care activities.^28,29,35,37,50^ In these, professional interpreting increased in some but not all of the specific clinical interactions assessed.^28,29,35,37,50^

Two studies assessed patient report of always having professional interpreting.^41,61^ One study described increased professional interpreting.^41^ The other study found no change, despite an increase in clinician report of professional interpreting through EHR documentation.^61^

### Implementation Strategies

Most studies assessed intervention bundles (29 [74%]).^28,29,30,31,33,34,36,37,38,39,41,42,43,44,48,51,53,54,55,56,58,59,60,61,62,63,64,65,66,67^; only 10 studies (26%) included single-strategy interventions.^32,35,40,45,46,47,49,50,52,57^ Based on our initial review, we categorized implementation strategies as education, increasing access, standardized policies and procedures, modifications to the EHR, or other (those that did not fit into one of these categories). Improvements in professional interpreting were seen with all strategy types.^28,30,32,33,34,35,36,37,38,39,40,41,42,43,44,45,46,47,48,49,50,51,52,53,54,55,57,58,59,60,61,62,63,64,65,66,67^

#### Clinician Education

Twenty studies (51%) implemented a strategy involving education about professional interpreting.^28,29,30,32,37,38,39,41,42,43,45,48,50,53,55,56,58,59,62,65,66^ Mode of delivery varied and included education delivered at existing meetings,^28,29,41,48,56,60,61^ during clinical shifts,^42,55,66^ through online modules,^45,53,56^ and in materials available physically and electronically.^28,29,30,37,38,39,42,43,48,53,55,56,58,59,61,66^ Two studies incorporated skill building through role-playing or case-based activities.^32,53^

#### Increasing Access to Professional Interpreting

Eighteen studies (46%) implemented a strategy to increase access to professional interpreting.^28,29,34,37,40,41,43,46,47,48,53,55,58,60,61,62,64,65,66^ These strategies included hiring more staff interpreters,^34,48^ increasing technical bandwidth for remote access,^34,48,55^ increasing the number of remote interpreting devices available to clinicians,^40,46,48,53,61,64,66^ having a dedicated in-person interpreter present in clinical areas during high-need times,^60,62,65^ placing a remote interpreting device in all rooms^28,29,37,41,60^ or the rooms of patients using LOE,^43,58^ or downloading an application with a direct link to a telephone interpreting service.^47^

#### Policies and Procedures Related to Professional Interpreting

Twelve studies (31%) involved implementing a policy or procedure related to professional interpreting.^33,44,48,52,53,56,57,59,60,61,62,65^ Examples included the incorporation of patient language into daily safety huddles or patient handoffs,^44,62^ procedures to support the advanced scheduling and coordination of patient care activities with professional interpreter availability,^33,44,53,56,65^ and standardized procedures for the assessment of patient language and interpreter need during the initial patient interaction.^33,44,52,53,56,59,60,61,65^ A variety of approaches were used to assess patient language and interpreter need. One randomized trial compared 3 questions (“Would you like an interpreter?” “A free hospital interpreter is available for you. Would you like an interpreter?” and “In what language do you prefer to receive your medical care?”), and researchers found that professional interpreting occurred most frequently when patients were asked their preferred language for medical care.^52^ Five strategies assessed English language proficiency^61^ or preference,^33,44,53,65^ and 2 assessed clinician or parent perception of interpreter need.^44,56^ Two implementation strategies used a 2-level approach (assessing language then interpreting preferences).^59,60^

Four studies assessed changes in language identification after strategy implementation.^33,36,59,61^ Of these, 2 studies demonstrated improvement in the proportion of patients with language documented in the EHR,^33,36^ and 1 study demonstrated an increase in recognition of interpreter need during the initial clinical interaction.^59^

#### EHR Modifications

Fourteen studies (36%) implemented a strategy modifying the EHR.^33,39,41,51,53,54,55,58,59,60,62,63,65,67^ These modifications included an icon or field indicating language or interpreter need on patient lists,^51,55,58,59,60^ pop-up alerts about language need or interpreter need (or both),^41,59^ a computerized order for in-person interpreting,^53^ prompts with standardized scripts for assessing interpreter need,^33,59,65^ and templates for documenting professional interpreting.^51,53,58,59,63,65,67^ Two studies that implemented documentation templates built in hard stops preventing clinicians from finalizing documentation without completing a section related to professional interpreting.^63,67^

#### Other Implementation Strategies

Fifteen studies (39%) included 1 or more other implementation strategies.^31,33,35,39,44,48,49,53,55,58,60,62,64,65,66^ Four strategies included an assessment of clinicians’ LOE skills.^31,35,49,64^ Assessment occurred in several ways: 2 studies used a standardized oral language testing service to assess overall and medical language proficiency (including expression and comprehension),^35,64^ 1 study used an orally administered abbreviated Spanish health literacy examination,^49^ and 1 study used mock interviews, followed by a written test to assess comprehension before and after participation in a 20-hour medical Spanish course.^31^

Four studies included auditing professional interpreting data, shared with clinicians via email,^44,60^ in-person meetings,^48^ or a central data visualization dashboard.^60,65^ Three studies included the identification of a clinician language access champion,^33,39,55^ and 1 study partnered individual professional interpreting data with an existing program that provided financial incentives for participating physicians.^53^ Six studies indirectly targeted clinician behaviors through interpreter rounding or patient-facing materials (brochures, flyers, videos) to encourage or facilitate patient requests for professional interpreting.^33,48,58,62,65,66^

### Implementation Strategies Mapped to the COM-B Model and the TDF

Mapped to the COM-B model, implementation strategies targeted clinician capability (32 interventions [82%]),^28,29,30,32,33,37,38,39,41,42,43,44,45,47,48,50,52,53,55,56,58,59,60,61,62,63,65,66,67^ opportunity (28 [72%]),^28,29,33,34,37,39,40,41,43,44,46,47,48,51,52,53,55,56,57,58,59,60,61,62,63,64,65,66,67^ and motivation (22 [56%]).^30,31,32,33,35,38,39,41,44,45,48,49,51,52,53,55,56,58,59,60,61,62,63,64,65,66,67^ Most strategies (30 [77%]) targeted more than 1 COM-B domain.^28,29,30,32,33,36,37,38,39,41,43,44,45,47,48,51,52,53,54,55,56,58,59,60,61,62,63,64,65,66,67^ Table 5 shows COM-B and TDF targets for each implementation strategy type, as detailed hereinafter.

**Table 5.** Implementation Strategies Mapped to COM-B Barriers to Professional Interpreting, With TDF Domain

Strategy to enact behavior change	No. (%) of studies using the strategy	COM-B target and TDF domain^a^		
		Capability barriers	Opportunity barriers	Motivation barriers
Education				
How and when to access language services	20 (51)	Technical knowledge	NA	NA
Why use professional interpreters	15 (38)	Conceptual knowledge	NA	Beliefs about consequences; social and professional role/identity; emotions
Hands-on scenario-based practice working with professional interpreters	2 (5)	Skills	NA	Beliefs about capabilities; beliefs about consequences; social and professional role/identity; emotions; optimism; reinforcing behavior
Increasing access to professional interpreting				
Device for connection to remote interpreting in all patient rooms or in all rooms of patients using LOE	Telephone: 6 (15); video: 1 (3)	Memory, attention, decision processes	Environmental context and resources; social influences	NA
Increasing number of remote interpreting devices for general access	Telephone: 2 (5); video: 6 (16)	Memory, attention, decision processes	Environmental context and resources	NA
Dedicated in-person interpreter present in patient care areas during high-need times	3 (8)	Memory, attention, decision processes	Environmental context and resources; social influences	NA
Increasing technical bandwidth	3 (8)	NA	Environmental context and resources	NA
Hiring more staff interpreters	2 (5)	NA	Environmental context and resources	NA
App on clinician- or hospital-owned device with direct link to telephone interpreting service	1 (3)	Memory, attention, decision processes	Environmental context and resources	NA
Policies and procedures				
Standardized process for assessing language and interpreter need	9 (23)	Memory, attention, decision processes	Environmental context and resources	Beliefs about consequences; social and professional role/identity; emotions
Standardized process for advance scheduling of professional interpreters (eg, during rounds or outpatient visits)	5 (13)	Memory, attention, decision processes	Environmental context and resources; social influences	NA
Standardized policy prohibiting ad hoc interpreters	1 (3)	NA	Environmental context and resources; social influences	Beliefs about consequences; social and professional role/identity; emotions
Standardized process for including language in daily safety huddles or patient handoffs	2 (5)	NA	Environmental context and resources; social influences	Social and professional role/identity; emotions; goals; intentions
Standardized policies promoting telephone communication in place of in-person interactions during early COVID-19 pandemic	1 (3)	NA	Environmental context and resources; social influences	NA
EHR modifications				
Icon indicating patient language on patient lists	5 (13)	Memory, attention, decision processes	Environmental context and resources	Beliefs about consequences; social and professional role/identity; emotions
Standardized phrase for documentation of professional interpreting entered into clinician note at clinician discretion	4 (10)	NA	Environmental context and resources	Social and professional role/identity
EHR-based script for standard assessment of language and interpreter need	3 (8)	Memory, attention, decision processes	Environmental context and resources	Beliefs about consequences; social and professional role/identity; emotions
Pop-up alert about language and/or professional interpreting in medical records of patients using LOE	2 (5)	Memory, attention, decision processes	Environmental context and resources	Beliefs about consequences; social and professional role/identity
Hard stop in clinician note for all patients requiring documentation of professional interpreting	2 (5)	Memory, attention, decision processes	Environmental context and resources	NA
Optional selection documenting professional interpreting in clinician notes for all patient records	1 (3)	Memory, attention, decision processes	Environmental context and resources	Beliefs about consequences; social and professional role/identity
Section for documentation of professional interpreting in clinician notes automatically populated in medical records for patient using LOE	1 (3)	Memory, attention, decision processes	Environmental context and resources	NA
Field for language automatically added to daily progress notes	1 (3)	Memory, attention, decision processes	Environmental context and resources	Beliefs about consequences; social and professional role/identity; emotions
Field for language added to patient medical record	1 (3)	Memory, attention, decision processes	Environmental context and resources	Beliefs about consequences; social and professional role/identity; emotions
EHR order for in-person interpreting	1 (3)	Memory, attention, decision processes	Environmental context and resources	NA
Other				
Patient-facing materials to facilitate request for interpreting	6 (15)	Memory, attention, decision processes	Environmental context and resources; social influences	Social and professional role/identity; intentions
Professional interpreting use data audit and feedback	4 (10)	NA	Social influences	Beliefs about consequences; social and professional role/identity; emotions; reinforcing behavior
Language skills or proficiency testing	4 (10)	NA	NA	Beliefs about capabilities
Staff champion(s)	3 (8)	NA	Social influences	NA
Language for care sign on door to patient rooms	1 (3)	Memory, attention, decision processes	Environmental context and resources	Beliefs about consequences; social and professional role/identity; emotions
Financial incentives	1 (3)	NA	NA	Reinforcing behavior

Abbreviations: COM-B, Capability, Opportunity, Motivation–Behavior; EHR, electronic health record; LOE, languages other than English for medical care; NA, not applicable; TDF, Theoretical Domains Framework.

^a^ See also Table 1.

#### Capability

All education strategies targeted clinicians’ capability to partner with professional interpreters by supporting clinicians’ technical knowledge of how to access professional interpreting.^28,29,30,32,37,38,39,41,42,43,45,48,50,53,55,56,58,59,62,65,66^ Fifteen strategies also provided conceptual knowledge regarding how professional interpreting improves patient care,^30,32,38,39,41,45,48,53,55,56,58,60,62,65,66^ and 2 incorporated skill-building activities.^32,53^ All other strategies targeting capability did so by decreasing the memory, attention, and decision-making processes (ie, cognitive load) required to identify language, access professional interpreting, or both.^28,29,33,37,39,41,43,44,47,48,51,52,53,55,56,58,59,60,61,62,63,65,66,67^ Examples include strategies that made professional interpreting readily available (eg, always at patient bedside^28,29,37,41,43,58,60^ or present in-person during high-need times,^60,62,65^ via direct link on clinician devices,^47^ or via advance scheduling^33,44,53,56,65^) and strategies that facilitated clinician recognition of language or interpreter need (eg, notification during patient safety huddles^44,62^ or icons and pop-up alerts in the EHR^41,51,55,58,59,60^). Patient-facing materials indirectly decreased clinician cognitive load by increasing patient-led opportunities to reinforce professional interpreting.^33,48,58,62,65,66^

#### Opportunity

Strategies that targeted clinicians’ opportunities to partner with professional interpreters often did so by modifying the environmental context.^28,29,33,34,37,39,40,41,43,44,46,47,48,51,52,53,55,56,57,58,59,60,61,62,63,64,65,66,67^ These strategies included increasing access to professional interpreting^28,29,34,37,40,41,43,46,47,48,53,55,58,59,60,61,62,64,65,66^ and modifications to the EHR^33,39,41,51,53,54,55,58,59,60,62,63,65,67^ (ie, modifications to the physical environment), as well as the development of policies and procedures for language access (ie, modifications to the care delivery system).^33,44,48,52,53,56,57,59,60,61,62,65^ Several strategies additionally targeted clinician opportunity by providing a social influence by fostering a culture that normalized professional interpreting.^28,29,33,37,39,43,44,48,53,55,56,57,58,60,62,65,66^ Examples included the placement of a remote interpreting device at all patient bedsides,^28,29,37,41,60^ the physical presence of an in-person interpreter in patient care areas during high-need times^60,62,65^ or as scheduled in advance,^33,44,53,56,65^ sharing overall professional interpreting use data to clinicians,^44,48,60,65^ identification of a staff language access champion,^33,39,55^ and use of patient-facing materials to facilitate patient requests for professional interpreting.^33,48,58,62,65,66^

#### Motivation

Two implementation strategies directly targeted clinician motivation to partner with professional interpreters: testing of clinician language skills or proficiency,^31,35,49,64^ which targeted clinicians’ beliefs about their capabilities to communicate without professional interpreting, and use of financial incentives,^53^ which provided reinforcing behaviors. Other strategies targeted motivation indirectly. For example, educational sessions that included skill-building targeted clinicians’ motivation by enhancing beliefs about their capabilities, and they also provided optimism around clinicians’ ability to partner with professional interpreters and reinforcing behaviors through positive feedback from successfully applying newly learned skills.^32,53^ Several strategies indirectly targeted motivation by providing reinforcement that supported clinicians’ beliefs about the consequences of communicating with and without professional interpreting, perceptions of their social and professional identity around providing high-quality care for patients, and potential negative emotions associated with getting by without professional interpreting. These strategies included education presenting conceptual knowledge about the rationale for professional interpreting,^30,32,38,39,41,45,48,53,55,56,58,60,62,65,66^ policies prohibiting ad hoc interpreting,^48^ use of patient-facing materials that encouraged patient requests for professional interpreting,^33,48,58,62,65,66^ and reminders of patient language in the EHR,^33,41,51,55,58,59,60,65^ on patient room doors,^60^ in patient handoffs,^44,62^ and through sharing overall professional interpreting use data to clinicians.^44,48,60,65^

## Discussion

A wide range of targeted clinician behavior change strategies have been implemented across clinical settings, with the aim of increasing professional interpreting. In this review, we identified high heterogeneity in strategies and outcome assessment and observed that professional interpreting often remained suboptimal, even after implementation. Taken together with the overall high risk of bias, this high heterogeneity prevented us from directly identifying which strategies are most effective. However, our findings highlight current gaps and areas for improving language equity. With this, we set a focused agenda for clinicians and researchers to move forward.

There are important challenges to ensuring language access. For example, as evident by the myriad approaches identified in this review, there is no evidence-based best practice for assessing interpreter need that has been demonstrated to be valid, patient centered, and responsive to context. In addition to the implications for clinical communication with patients, inconsistencies in assessing language hamper health systems approaches to improving care. Additionally, the wide variability in outcome ascertainment demonstrates the difficulty in accurately assessing whether professional interpreting occurs when necessary. Although systematically collected administrative data are an objective measure, they may only capture the minimum standard (that professional interpreting occurred). However, in many settings, a single interaction between a clinician and a patient is unlikely.^14^ Through administrative data, we can only identify those instances in which professional interpreting occurred; we cannot identify communication occurring without professional interpreting. Similarly, clinician EHR documentation of professional interpreting may not be linked to discrete communication events, limiting the ability to determine whether professional interpreting occurred during all interactions. Clinician- and patient-reported measures are subject to recall and social desirability bias. Direct observation provides objective, rich, and reliable data but may not be feasible in many circumstances.^13,14^ These limitations must be addressed to rigorously advance the science of language equity.

Thus, there are important next steps for health systems, clinicians, and researchers. First, we must rigorously develop reliable and patient-centered approaches to assessing language and interpreter need. Assessing English proficiency, as recommended by the Institute of Medicine and used by at least 1 study in this review, is a valid measurement that is well suited for research^68,69^; however, this approach is deficit focused and may be off-putting to patients.^19,20^ Instead, we recommend a 2-level approach, as used by Hartford et al^60^ and Gupta et al^59^ at the first point of patient contact. With this approach, the patient is first asked if they use LOE (“What language would you like for care today?”^60^ or “Do you speak any languages other than English?”^59^); for those who do, a follow-up statement is made about the availability of free professional interpreting services and a question assessing the patient’s interest in or need for such is asked (“Can we provide free interpretation in [language of choice]?”^60^ or “Our hospital has free and quickly accessible interpreter services. What language would you like us to speak to you in?”^59^). Importantly, these recommendations require rigorous testing to establish patient-centeredness, sensitivity, and specificity. Furthermore, given that language proficiency is context dependent,^19^ we must develop processes to continuously reassess interpreting need across and within patient encounters.

Second, we must also establish best practices in outcome measurement. For studies conducted in outpatient settings, in which most communication occurs in a single interaction, administrative data to assess the occurrence of professional interpreting at least once per visit are likely sufficient but may require system changes to connect overall interpreter billing data to patient records for monitoring and audit purposes. However, for clinical encounters with fragmented communication, as in emergency department and inpatient settings, objective administrative data should be partnered with patient-reported measurements. This approach allows investigators to triangulate their findings to address the limitations of different outcome types and gain a more nuanced understanding of their local professional interpreting landscape.

Our findings demonstrate a clear need for rigorous research to identify which implementation strategies, in which settings, are most effective at increasing professional interpreting. By mapping implementation strategies to the COM-B and the TDF, our results support a hypothesis-driven process in which leaders can identify salient local barriers to professional interpreting and target those with specific implementation strategies. However, our continued inability to assess the comparative effectiveness of different strategies is a critical gap for organizations with limited resources and likely prevents the widescale adoption of such strategies. Future research should test individual strategies with multifactorial study designs to identify the minimum behavior change components needed to effectively increase professional interpreting based on local contextual factors, while simultaneously evaluating sustainment and long-term outcomes.

### Limitations

This systematic review is subject to limitations. Despite a rigorous approach, it is possible we missed studies. Our findings are subject to publication bias, because we only included studies that had undergone peer review and we did not search the gray literature. Because many negative studies are not published, we may be overestimating positive outcomes. We also chose to include studies written in English, regardless of their country of origin. Although this allowed us to include a broader range of studies, health care systems across countries vary, which may affect how and when professional interpreting occurs. Furthermore, we may have missed studies written in LOE that otherwise met our inclusion criteria.

## Conclusions

In this systematic review, a wide range of targeted behavior change strategies were implemented to increase professional interpreting, albeit with substantial heterogeneity, risks for bias, and persistent gaps in professional interpreting. Moving forward, we need rigorous, multifactorial interventional investigations that will identify the minimum behavior change components—based on local contextual factors—needed to effectively increase professional interpreting for all communication with patients using LOE.

## Notes

Supplement 1.
eMethods
**eTable.** Adapted Tool to Assess Study Quality **eFigure.** PRISMA Flow Diagram

Supplement 2.
Data Sharing Statement
