# Supplementary material for: Strategies to Increase Professional Interpreting in Clinical Settings: A Systematic Review
Source: JAMA Netw Open. 2025 Jul 17;8(7):e2521492. doi: 10.1001/jamanetworkopen.2025.21492 (PMC12272290; doi:10.1001/jamanetworkopen.2025.21492)
Supplement: Supplement 1. — eMethods eTable. Adapted Tool to Assess Study Quality eFigure. PRISMA Flow Diagram [file jamanetwopen-e2521492-s001.pdf]

Supplemental Online Content

Gutman CK, Rojas CR, Waidner L, et al. Strategies to increase professional interpreting in clinical settings: a systematic review. *JAMA Netw Open*. 2025;8(7):e2521492. doi:10.1001/jamanetworkopen.2025.21492

eMethods

eTable. Adapted Tool to Assess Study Quality

eFigure. PRISMA Flow Diagram

This supplemental material has been provided by the authors to give readers additional information about their work.

© 2025 Gutman CK et al. *JAMA Network Open*.

eMethods

Details of Search Strategy

Keyword synonyms for search concepts

concept	patients/caregivers who use a language other than English	interpreting services
synonyms	communication barrier(s)<br>English as a second language<br>English language proficiency<br>foreign language(s)<br>language barrier(s)<br>language for care<br>language(s) other than English<br>language preference(s)<br>language proficiency<br>limited English proficiency<br>non-English<br>preferred language(s)	interpreter(s)<br>interpreting<br>translating<br>translation(s)<br>translator(s)

Eligible studies used to validate PubMed strategy

Cheston CC, Alarcon LN, Martinez JF, Hadland SE, Moses JM. Evaluating the Feasibility of Incorporating In-Person Interpreters on Family-Centered Rounds: A QI Initiative. *Hosp Pediatr*. 2018;8(8):471-478. doi:10.1542/hpeds.2017-0208

Gupta KM, Campeggio D, Madu C, et al. Improving Identification of Interpreter Need in the Pediatric Emergency Department. Pediatrics. 2023;151(3):e2022057330. doi:10.1542/peds.2022-057330

Hartford EA, Rutman LE, Fenstermacher S, et al. Improving and Sustaining Interpreter Use Over 5 Years in a Pediatric Emergency Department. *Pediatrics*. Published online May 18, 2023:e2022058579. doi:10.1542/peds.2022-058579

Lion KC, Ebel BE, Rafton S, Zhou C, Hencz P, Mangione-Smith R. Evaluation of a Quality Improvement Intervention to Increase Use of Telephonic Interpretation. Pediatrics. 2015;135(3):e709-e716. doi:10.1542/peds.2014-2024

Rajbhandari P, Keith MF, Braidy R, Gunkelman SM, Smith E. Interpreter Use for Limited English Proficiency Patients/Families: A QI Study. Hosp Pediatr. 2021;11(7):718-726. doi:10.1542/hpeds.2020-003889

Tuot DS, Lopez M, Miller C, Karliner LS. Impact of an Easy-Access Telephonic Interpreter Program in the Acute Care Setting: An Evaluation of a Quality Improvement Intervention. Jt Comm J Qual Patient Saf. 2012;38(2):81-AP21. doi:10.1016/S1553-7250(12)38011-2

Search Strategies

All searches were performed on July 28, 2023 and then again on September 2, 2024.

<u>CINAHL (n=2701)</u>
Coverage: EBSCOhost Research Databases, 1981 onward
Search date: July 28, 2023

#	Query	Results
S1	(MH "Communication Barriers+")	6,131
S2	TX communication N3 barrier*	7,688
S3	TX English N3 (“other than” OR proficiency OR “second language”)	5,368
S4	TX (barrier* OR care OR foreign OR preference* OR preferred OR proficiency) N3 language*	7,357
S5	TX non-English	2,177
S6	S1 OR S2 OR S3 OR S4 OR S5	19,512
S7	(MH "Medical Interpretation Services")	31

© 2025 Gutman CK et al. *JAMA Network Open*.

S8	TX interpreter* OR interpreting OR translating OR translation* OR translator*	120,822
S9	S7 OR S8	120,831
S10	S6 AND S9<br><br>Limiters - English Language<br>Expanders - Apply equivalent subjects<br>Search modes - Boolean/Phrase	2,701

CINAHL (n=162)
Coverage: EBSCOhost Research Databases, 1981 onward
Search date: September 2, 2024

#	Query	Results
S1	(MH "Communication Barriers+")	6,548
S2	TX communication N3 barrier*	8,116
S3	TX English N3 (“other than” OR proficiency OR “second language”)	5,654
S4	TX (barrier* OR care OR foreign OR preference* OR preferred OR proficiency) N3 language*	7,907
S5	TX non-English	2,299
S6	S1 OR S2 OR S3 OR S4 OR S5	20,665
S7	(MH "Medical Interpretation Services")	43
S8	TX interpreter* OR interpreting OR translating OR translation* OR translator*	62,182
S9	S7 OR S8	62,193
S10	S6 AND S9<br><br>Limiters - Publication Date: 20230701-20241231; English Language<br>Expanders - Apply equivalent subjects<br>Search modes - Proximity	162

Embase.com (n=3253)
Coverage: Embase, MEDLINE, Preprints; 1974 onward
Search date: July 28, 2023

('communication barrier'/de OR 'english proficiency'/exp OR ((communication NEAR/3 barrier*):ti,ab,kw) OR ((english NEAR/3 ('other than' OR proficiency OR 'second language')):ti,ab,kw) OR (((barrier* OR care OR foreign OR preference* OR preferred OR proficiency) NEAR/3 language*):ti,ab,kw) OR 'non english':ti,ab,kw) AND ('interpreter service'/de OR interpreter*:ti,ab,kw OR interpreting:ti,ab,kw OR translating:ti,ab,kw OR translation*:ti,ab,kw OR translator*:ti,ab,kw) AND [english]/lim

Embase.com (n=621)
Coverage: Embase, MEDLINE, Preprints; 1974 onward
Search date: September 2, 2024

('communication barrier'/de OR 'english proficiency'/exp OR ((communication NEAR/3 barrier*):ti,ab,kw) OR ((english NEAR/3 ('other than' OR proficiency OR 'second language')):ti,ab,kw) OR (((barrier* OR care OR foreign OR preference* OR preferred OR proficiency) NEAR/3 language*):ti,ab,kw) OR 'non english':ti,ab,kw) AND ('interpreter service'/de OR interpreter*:ti,ab,kw OR interpreting:ti,ab,kw OR translating:ti,ab,kw OR translation*:ti,ab,kw OR translator*:ti,ab,kw) AND [english]/lim AND [01-07-2023]/sd NOT [31-12-2024]/sd

© 2025 Gutman CK et al. *JAMA Network Open*.

Ovid MEDLINE (n=2813)
Coverage: (R) ALL, 1946 to July 27, 2023
Search date: July 28, 2023

1	exp Communication Barriers/ or exp Limited English Proficiency/	7569
2	(communication adj3 barrier*).ab,kf,ti.	2703
3	(English adj3 (other than or proficiency or second language)).ab,kf,ti.	4547
4	((barrier* or care or foreign or preference* or preferred or proficiency) adj3 language*).ab,kf,ti.	9257
5	non-English.ab,kf,ti.	4195
6	1 or 2 or 3 or 4 or 5	24005
7	exp Translating/	5886
8	(interpreter* or interpreting or translating or translation* or translator*).ab,kf,ti.	363144
9	7 or 8	365853
10	6 and 9	2943
11	limit 10 to english language	2813

Ovid MEDLINE (n=363)
Coverage: (R) ALL, 1946 to August 29, 2024
Search date: September 2, 2024

1	exp Communication Barriers/ or exp Limited English Proficiency/	7860
2	(communication adj3 barrier*).ab,kf,ti.	3093
3	(English adj3 (other than or proficiency or second language)).ab,kf,ti.	5209
4	((barrier* or care or foreign or preference* or preferred or proficiency) adj3 language*).ab,kf,ti.	10621
5	non-English.ab,kf,ti.	4859
6	1 or 2 or 3 or 4 or 5	26831
7	exp Translating/	6095
8	(interpreter* or interpreting or translating or translation* or translator*).ab,kf,ti.	392816
9	7 or 8	395556
10	6 and 9	3305
11	limit 10 to english language	3169
12	limit 11 to dt=20230701-20241231	363

PubMed (n=2599)
Coverage: PubMed-Not-Medline and PubMed In-Process, 1946 onward
Search date: July 28, 2023

("communication barriers"[MeSH Terms] OR "limited English proficiency"[MeSH Terms] OR "communication barrier*"[Text Word] OR "English as a second language"[Text Word] OR "English language proficiency"[Text Word] OR "foreign language*"[Text Word] OR "language barrier*"[Text Word] OR "language other than english"[Text Word] OR "language proficiency"[Text Word] OR "limited English proficiency"[Text Word] OR "non-English"[Text Word] OR "preferred language*"[Text Word]) AND ("translating"[MeSH Terms] OR "interpreter*"[Text Word] OR "interpreting"[Text Word] OR "translating"[Text Word] OR "translation*"[Text Word] OR "translator*"[Text Word]) AND "english"[Language]

PubMed (n=341)
Coverage: PubMed-Not-Medline and PubMed In-Process, 1946 onward
Search date: September 2, 2024

(("communication barriers"[MeSH Terms] OR "limited English proficiency"[MeSH Terms] OR "communication barrier*"[Text Word] OR "English as a second language"[Text Word] OR "English language proficiency"[Text Word] OR "foreign language*"[Text Word] OR "language barrier*"[Text Word] OR "language other than english"[Text Word] OR "language proficiency"[Text Word] OR "limited English proficiency"[Text Word] OR "non-English"[Text Word] OR "preferred language*"[Text Word]) AND ("translating"[MeSH Terms] OR "interpreter*"[Text Word] OR "interpreting"[Text Word] OR "translating"[Text Word] OR "translation*"[Text Word] OR "translator*"[Text Word]) AND "english"[Language]) AND (2023/7/1:2024/12/31[pdat])

Web of Science (n=4539)
Coverage: WOS, 1985 onward; MEDLINE, 1950 onward; SCIELO, 2002 onward
Search date: July 28, 2023

#1 communication NEAR/3 barrier* (Topic) OR English NEAR/3 (“other than” OR proficiency OR “second language”) (Topic) OR (barrier* OR care OR foreign OR preference* OR preferred OR proficiency) NEAR/3 language* (Topic) OR

© 2025 Gutman CK et al. *JAMA Network Open*.

non-English (Topic)
Results: 30015

#2 interpreter* OR interpreting OR translating OR translation* OR translator* (Topic)
Results: 1330863

Search: #2 AND #1 and English (Languages)
Results: 4539

Web of Science (n=560)
Coverage: WOS, 1985 onward; MEDLINE, 1950 onward; SCIELO, 2002 onward
Search date: September 2, 2024

Search: ((((TS=(communication NEAR/3 barrier*)) OR TS=(English NEAR/3 ("other than" OR proficiency OR "second language"))) OR TS=((barrier* OR care OR foreign OR preference* OR preferred OR proficiency) NEAR/3 language*)) OR TS=(non-English)) AND TS=(interpreter* OR interpreting OR translating OR translation* OR translator*) and English (Languages)

Timespan: 2023-07-01 to 2024-12-31
Results: 560

© 2025 Gutman CK et al. *JAMA Network Open*.

eTable. Adapted Tool to Assess Study Quality

Item	Source	Bias Type Assessed
Is there a clearly stated aim?[1]	MINORS[21]	-
Are the study subjects and setting described in detail?[1]	JBI Cross-Sectional Study CAT	-
Is baseline access to and use of professional interpreting described in detail?[1]	QI-MQCS[22]	-
Is at least one implementation strategy described in detail?[1]	QI-MQCS	-
Are inclusion criteria defined?[1]	JBI Cross-Sectional Study CAT	Sampling
Were data collected prospectively?[2]	MINORS	Recall
Were participants enrolled consecutively?[3]	MINORS	Selection
Is the study participation rate reported?[4]	--	Selection, nonresponse
Is the outcome measure valid?[5]	JBI Quasi-Experimental Study CAT	Recall, social desirability
Are participants similar across comparisons?[6]	JBI Quasi-Experimental Study CAT	Unmeasured confounding
Is the analysis appropriate for the study question?[1]	JBI Critical Appraisal Tools, MINORS	Confirmation

MINORS – Methodological index for non-randomized studies; QI-MQCS – Quality improvement minimum quality criteria set; CAT – Critical appraisal tool
[1] Option responses are yes or no
[2] Option responses are yes, no, unclear, or not applicable (for cross-sectional study designs)
[3] Option responses are yes, no, unclear, or not applicable (if outcomes are not related to patient encounters)
[4] Option responses are yes, no, or not applicable (for administrative data, select yes unless the data presented is a raw number of interpreting encounters without a denominator or sample size)
[5] Option responses are yes (if administrative data with an associated sample size or patient or clinician report at or near the time of a specific clinical encounter) or no (if clinician report of general practice patterns or if administrative data is presented without a denominator or sample size)
[6] Option responses are yes, no, or unclear

© 2025 Gutman CK et al. *JAMA Network Open*.

eFigure. PRISMA Flow Diagram

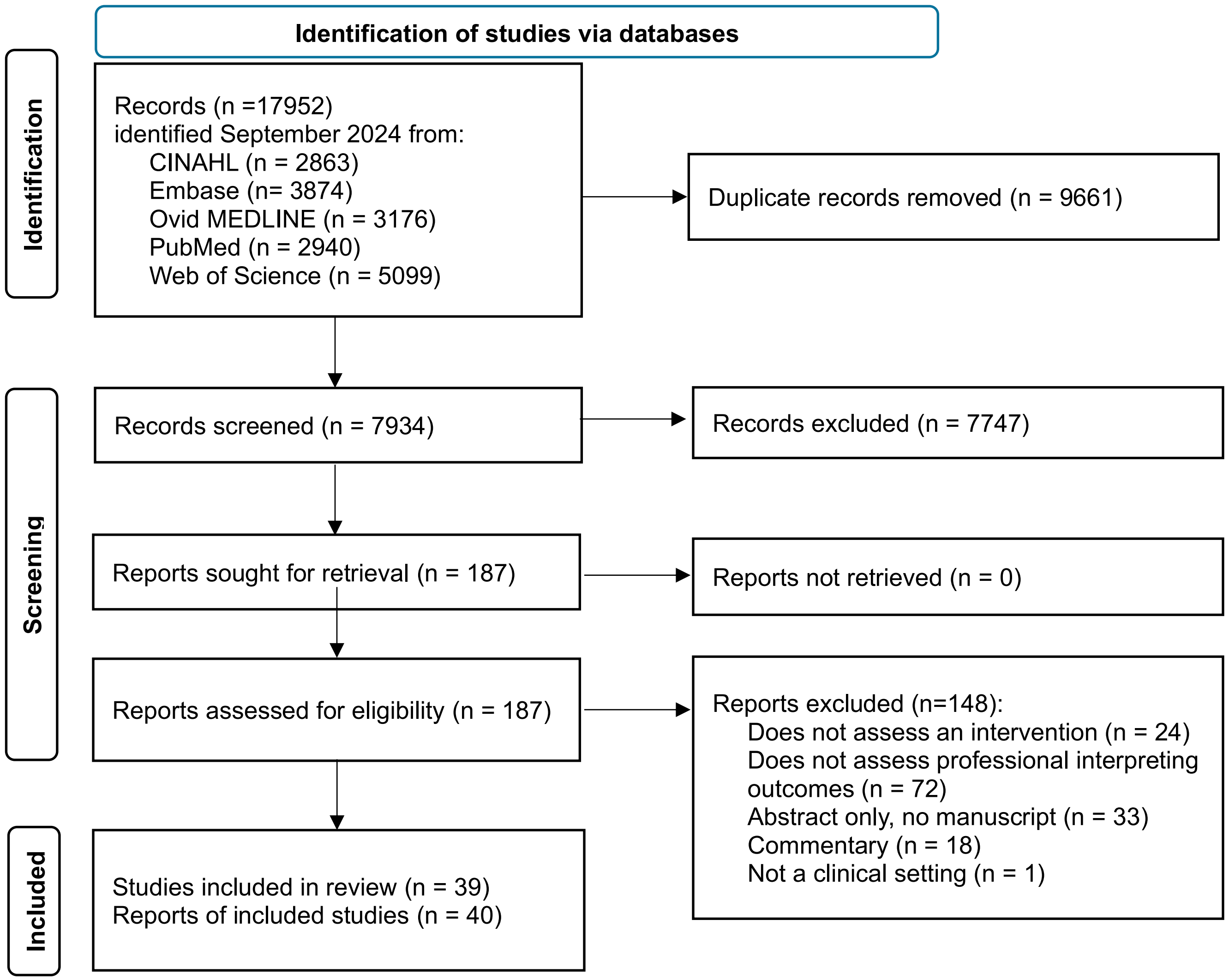

© 2025 Gutman CK et al. *JAMA Network Open*.
