# Supplementary material for: Strategies to Increase Professional Interpreting in Clinical Settings: A Systematic Review
Source: JAMA Netw Open. 2025 Jul 17;8(7):e2521492. doi: 10.1001/jamanetworkopen.2025.21492 (PMC12272290; doi:10.1001/jamanetworkopen.2025.21492)
Supplement: Supplement 2. — Data Sharing Statement [file jamanetwopen-e2521492-s002.pdf]

Data Sharing Statement

Gutman. Strategies to Increase Professional Interpreting in Clinical Settings. *JAMA Netw Open*. Published July 17, 2025. doi:10.1001/jamanetworkopen.2025.21492

Data
Data available: Yes
Data types: Data (not involving human participants)
How to access data: Data request can be sent to corresponding author
When available: With publication

Supporting Documents
Document types: None

Additional Information
Who can access the data: Researchers whose proposed use of the data has been approved
Types of analyses: secondary or confirmatory analyses
Mechanisms of data availability: approval of proposal, with a signed data access agreement
